# Clinical pharmacological study of dotinurad administered to male and female elderly or young subjects

**DOI:** 10.1007/s10157-019-01836-0

**Published:** 2019-12-30

**Authors:** Hiroshi Nakatani, Masahiko Fushimi, Tomomitsu Sasaki, Daisuke Okui, Tetsuo Ohashi

**Affiliations:** 1 Kitasato University Kitasato Institute Hospital Department of Research, Clinical Trial Center, 5-9-1 Shirokane, Minato-ku, Tokyo, 108-8642 Japan; 2 Development Department, Medical R&D Division, Fuji Yakuhin Co. Ltd., 4-383, Sakuragi-cho, Omiya-ku, Saitama-shi, Saitama, 330-9508 Japan

**Keywords:** Dotinurad, Pharmacokinetics, Pharmacodynamics, Selective urate reabsorption inhibitor, Age, Gender

## Abstract

**Background:**

Dotinurad is a novel selective urate reabsorption inhibitor (SURI) that selectively inhibits the reabsorption of uric acid in renal tubules and promotes the excretion of uric acid into urine. In this study, the effects of age and gender on the pharmacokinetics (PK), pharmacodynamics (PD), and safety of dotinurad were evaluated in healthy subjects.

**Methods:**

An open-label study of a single oral administration of dotinurad 1 mg was conducted in elderly (≥ 65 years) Japanese males and females, and young (20–35 years) males and females (six patients each).

**Results:**

Following a single-dose administration of dotinurad, the change in dotinurad plasma concentration showed a similar profile across groups. Regarding the PK parameters, there was no significant difference between elderly and young subjects. On comparing males and females, significant differences were observed in some parameters in elderly subjects. However, these differences in some parameters could not be detected by adjust for body weight. When PD parameters in elderly and young subjects were compared, significant differences were observed in some parameters in male subjects. On comparing males and females, significant differences were observed in some parameters in young subjects; however, the percent change in serum uric acid concentration decreased over time was relatively close for both groups. There were no clinically relevant safety problems.

**Conclusion:**

Age and gender had no clinically meaningful effect on the PK, PD, and safety of dotinurad.

**Clinical trials:**

ClinicalTrials.gov identifier: NCT02344875.

## Introduction

In Japan, hyperuricemia is defined as a serum uric acid level > 7.0 mg/dL and is considered to be a cause of urate crystal deposition diseases such as gouty arthritis and renal impairment [1]. In recent years, with the westernization of dietary habits, the number of hyperuricemic patients with or without gout has steadily been increasing. Currently, gout and hyperuricemia are considered to be lifestyle diseases comparable to hypertension, dyslipidemia, and diabetes mellitus [2, 3]. Correcting a high uric acid level and properly managing the serum uric acid level are important for the prevention of gouty arthritis and urinary calculi. Moreover, it was suggested that these actions may protect renal function and lower the risk of cardiovascular diseases in patients with gout and renal dysfunction [4, 5]. Therefore, from the perspective of preventing these diseases, we believe that the treatment of hyperuricemia will become more important in the future.

There are two types of antihyperuricemic: xanthine oxidase inhibitors and uricosuric drugs. The following are known causes of hyperuricemia: overproduction of uric acid “overproduction type”, decreased uric acid excretion “underexcretion type”, and a combination of both “combined type”. In Japan, prevalence of each is estimated to be 10%, 60%, and 30%, respectively. The second edition of the Japanese management guidelines recommended treatment based on the hyperuricemic type, as follows: “underexcretion type” is in principle treated with uricosuric drugs and “overproduction type” is treated with uric acid synthesis inhibitors [6]. The third edition of this guidelines, revised in December 2018, liberalized the basic principle of selecting drugs based on different type of hyperuricemia.

Dotinurad is a selective urate reabsorption inhibitor (SURI) that selectively inhibits urate transporter 1 (URAT1), expressed in renal proximal tubules. By inhibiting reabsorption of uric acid and promoting the excretion of uric acid into urine, dotinurad reduces the serum uric acid level [7].

In Japan, the age distribution of patients with gout shows that the largest group of patients is in their sixties [1]. Hyperuricemia and gout are common in elderly person, not to mention other adults. We can infer that the antihyperuricemics is one of the drugs commonly used in elderly patients. In general, many elderly persons have decreased physiological function involving organs such as the liver and kidneys. Therefore, when drugs are continuously administered, it is possible that maximum plasma concentration (*C*_max_) and area under the curve (AUC) are raised and unexpected adverse drug reactions occur due to delayed metabolism and excretion. For these reasons, we expect that, in some cases, dose reduction and adjustment of the dosing interval will be necessary in elderly patients.

A gender difference in serum uric acid concentration has been identified, generally higher in males. Thus, hyperuricemia is mostly in male patients; however, in female, the serum uric acid level increases after menopause [8].

We thus decided to examine the pharmacokinetics (PK), pharmacodynamics (PD), and safety of dotinurad in the elderly subjects, as well as gender difference in elderly males and females and young males and females.

## Methods

### Study design

This is a single-site, open-label study that examined the PK, PD, and safety of a single dose of dotinurad in elderly subjects, as well as gender-related differences.

Informed consent was obtained from all the subjects before the start of the study. Within 30 days before dotinurad administration, screening was completed including clinical examination, physical examination, vital signs, 12-lead electrocardiography (ECG), and clinical laboratory tests. The subjects who were judged eligible were assigned to one of the following four groups: elderly males aged ≥ 65 years, young males aged 20–35 years, elderly females aged ≥ 65 years, and young females aged 20–35 years. The subjects were hospitalized from the day before dotinurad administration to 2 days post-dose. Blood sampling and urine collection were conducted over time to monitor the subjects’ safety and to examine the PK and PD. On the next day of hospitalization, a single dose of dotinurad 1 mg was administered after a fast of at least 10 h. A follow-up examination was conducted within 5–10 days post-dose. During hospitalization, intake of the following was forbidden: all drugs, alcohol, tobacco, caffeine, grapefruit, and food containing St. John’s wort.

### Inclusion and exclusion criteria

The inclusion criteria were as follows: the subjects were Japanese; young males and females aged 20–35 years and elderly males and females aged ≥ 65 years at the time of informed consent obtained (six patients each group); body mass index (BMI) was 18.5–25.0 kg/m^2^; and a physician determined that they had no health problems at the screening.

The exclusion criteria were as follows: diseases of the digestive tract, heart, liver, and kidneys; a past history of surgery on the digestive tract or kidneys; a history of cancer, cerebral infarction, or myocardial infarction; a history of epileptic seizures or the possibility of inducing epileptic seizures because of an organic disorder of the brain; observation of a renal calculus on abdominal ultrasonography or plain abdominal radiography at the screening examination; an estimated glomerular filtration rate (eGFR) of less than 60 mL/min/1.73 m^2^; symptoms of allergy or idiosyncrasy to food, drugs, or metals as a current or historical condition; blood donation of at least 400 mL within 12 weeks before dotinurad administration; apheresis donation or blood donation of at least 200 mL within four weeks before dotinurad administration; use of any drug within 7 days before dotinurad administration; intake of alcohol within 3 days before dotinurad administration; intake of grapefruit or food containing grapefruit within 7 days before dotinurad administration; intake of St. John’s wort or food containing St. John’s wort within 4 weeks before dotinurad administration; administration of another study drug within 12 weeks of dotinurad administration; excessive exercise within 3 days before dotinurad administration; pregnancy or the possibility of pregnancy; breastfeeding; a desire to get pregnant or deliver during the study period; and assessment by a physician that the person was unsuitable as a subject of this study.

### Sample collection

Plasma, serum, and urine samples were collected to measure dotinurad, its major metabolites (glucuronide and sulfate conjugate), and uric acid concentration. Clinical trials in healthy adults have revealed that the main metabolites of dotinurad are glucuronide and sulfate conjugates (NCT02348307, NCT02901366). In a present study, it was considered useful to measure metabolites to investigate the pharmacokinetics of dotinurad in elderly subjects.

For PK, plasma samples were collected at the following times: before administration and at 0.5, 1, 2, 3, 4, 6, 8, 12, 24, 36, and 48 h post-dose. For PD, serum samples were collected at the following times: before administration and at 1, 2, 4, 6, 8, 12, 24, 36, and 48 h post-dose. Regarding urine samples, urine collection was conducted at the following periods: before administration (24 h before administration to immediately before administration) and during 0–6, 6–12, 12–24, and 24–48 h post-dose.

### Analytical methods

Plasma and urine concentrations of dotinurad and its major metabolites (glucuronide and sulfate conjugate) were measured using liquid chromatography (LC)–tandem mass spectrometry (MS/MS) at Fuji Yakuhin Co., Ltd.

For dotinurad concentration measurement: Agilent 1100 series (Agilent Technologies, USA) for LC, API3000 (AB SCIEX, USA) for MS, Inertsil ODS-3 (150 × 2.1 mm, 3 μm; GL Sciences, Inc., Japan) for column analyses, and 5 mmol/L ammonium acetate solution/methanol for the mobile phase. The limit of quantification was 1 ng/mL for the dotinurad concentration measurement in plasma and urine.

For dotinurad metabolite concentration measurement: Nexera X2, Prominence (Shimadzu, Japan) for LC, and Triple Quad 4500 (AB SCIEX) for MS. The limit of quantification was 1 ng/mL for dotinurad metabolite concentrations in plasma and urine, for both glucuronide conjugate and sulfate conjugate. Uric acid concentration in serum and urine was measured using the enzyme method, at the study site.

### Pharmacokinetics analyses

The PK parameters of dotinurad and its major metabolites (glucuronide and sulfate conjugates) were calculated for a non-compartment model using WinNonlin version 6.2. The main PK parameters used for PK evaluation were as follows: maximum plasma concentration (*C*_max_), time to reach the peak plasma concentration (*T*_max_), elimination half-life (*T*_1/2_), area under the plasma concentration–time curve from time zero to the last measurable sample point (AUC_0-t_) or from time zero to infinity (AUC_0-inf_), elimination rate constant (kel), distribution volume/fraction of dose absorbed (Vd/*F*), total clearance/fraction of dose absorbed (CL_tot_/F), mean residence time (MRT), amount of drug excreted in urine from time zero to 48 h (Ae_0-48_), and fraction of dose excreted in urine (fe). *C*_max_ and *T*_max_ were shown as actual measurement values of the drug concentration in plasma. AUC was estimated using a trapezoidal linear interpolation method available in WinNonlin. For the calculated PK parameters (*C*_max_, *T*_max_, *T*_1/2_, AUC_0-t_, and AUC_0-inf_), a *t* test was performed to compare elderly and young subjects, as well as males and females, to calculate the mean differences and their 90% confidence intervals.

### Pharmacodynamics analyses

The PD parameters of uric acid concentration in serum and urine were as follows: Delta maximum effective concentration (ΔEC_max_), delta area under the serum concentration–time curve from time zero to 48 h (ΔAUEC_0-48_), amount of drug excreted in urine from time zero to t hour (Ae_0-t_), renal clearance (CL_R_), and fractional uric acid excretion (FE). ΔAUEC_0-48_ was calculated from AUEC_–24–0_ and AUEC_0-48_. For the calculated PD parameters (ΔEC_max_, ΔAUEC_0-48_, and FE), a *t* test was performed to compare elderly and young, males and females, to calculate mean differences and their 90% confidence intervals.

### Safety evaluations

The clinical investigator evaluated safety based on adverse events (AEs), adverse drug reactions (ADRs), clinical laboratory test values, vital signs, and 12-lead ECG. AEs were classified according to the system organ class and preferred term (MedDRA version 17.0; Japanese Maintenance Organization, Tokyo, Japan) and were judged for potential causality in relation to dotinurad, severity, and seriousness by clinical investigator. AEs judged to be related to the study drug were defined as ADRs.

### Statistical analyses

All the statistical analyses were performed using SAS software (version 9.2.; SAS Institute Inc., Cary, NC, USA). The statistical tests and confidence intervals were two-sided and *P* < 0.05 denoted statistical significance.

## Results

### Subjects

Dotinurad was administered to six subjects in each group (elderly male, young male, elderly female, and young female), and no subjects were discontinued in this study. Table 1 shows the characteristics of the subjects who received dotinurad.

**Table 1:** Characteristics of subjects

	Elderly male (*n* = 6)	Young male (*n* = 6)	Elderly female (*n* = 6)	Young female (*n* = 6)
Age (years)^a^	71.3 ± 2.9	25.0 ± 4.6	69.3 ± 2.3	26.7 ± 4.4
(Minimum—maximum)	(67–75)	(20–32)	(66–72)	(23–34)
Body height (cm)	164.27 ± 6.83	170.88 ± 6.48	153.78 ± 3.45	158.73 ± 6.01
Body weight (kg)	59.03 ± 8.36	67.57 ± 5.32	52.97 ± 4.56	52.43 ± 8.62
BMI (kg/m^2^)	21.78 ± 2.29	23.08 ± 1.00	22.33 ± 1.36	20.63 ± 2.08
eGFR (mL/min/1.73m^2^)^b^	73.67 ± 10.34	107.67 ± 8.67	72.67 ± 4.19	95.67 ± 11.19
Serum uric acid concentration (mg/dL)	5.43 ± 1.20	6.00 ± 0.65	4.33 ± 0.66	4.33 ± 0.64

Each value is shown as mean ± SD

^a^Age of the consent acquisition day

^b^Post hoc analysis

### Pharmacokinetics

**Fig. 1:**
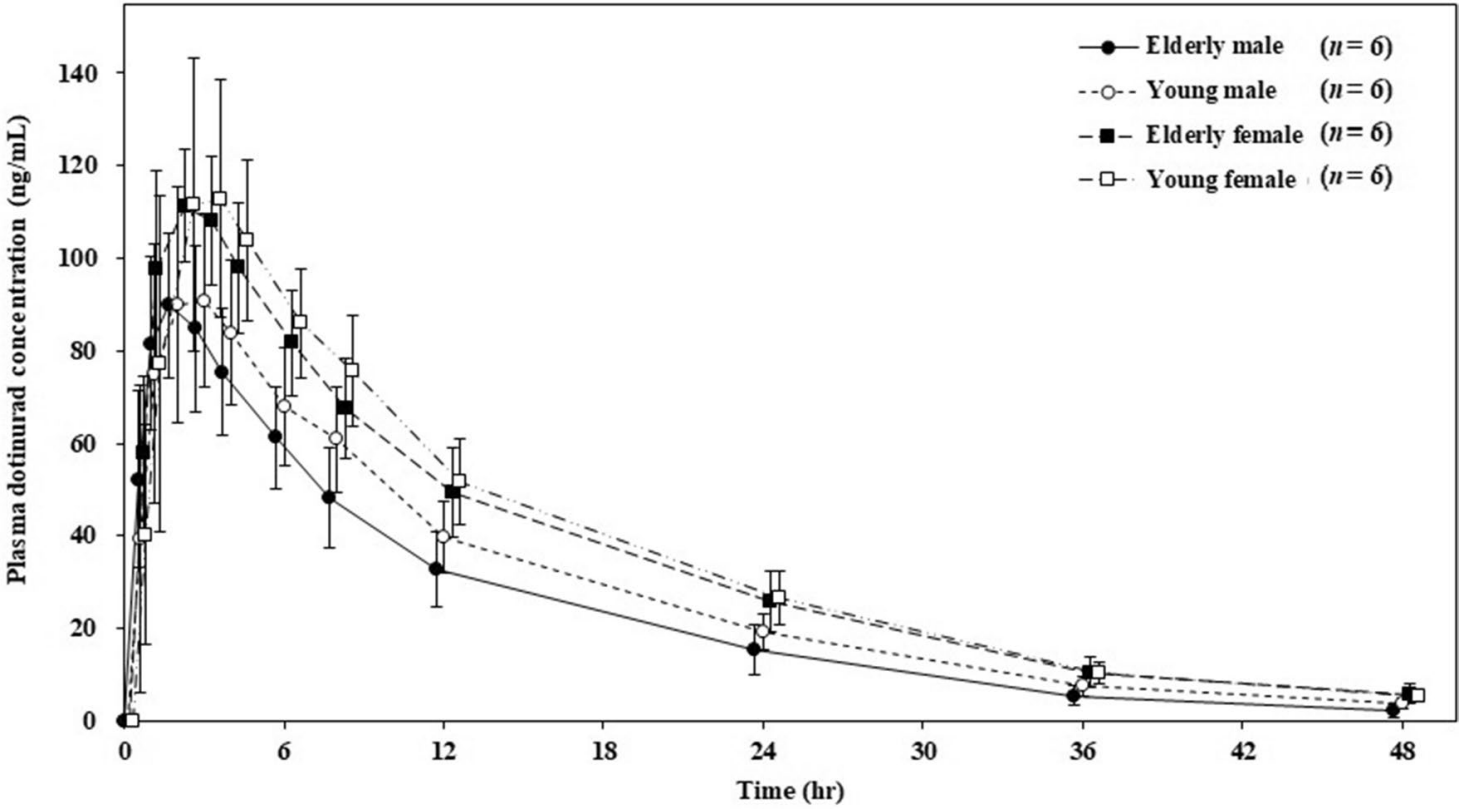
Time course of dotinurad concentrations. Error bars indicate standard deviation

Figure 1 shows the time course of plasma dotinurad concentrations in each group. The change of dotinurad concentration was in similar manner in each group. Table 2 shows the plasma and urine PK parameters of dotinurad. Overall, *T*_max_ was 2.00–2.83 h and *T*_1/2_ was 9.28–10.92 h. The other main plasma PK parameters of the elderly male, young male, elderly female, and young female groups were as follows: *C*_max_ was 93.30, 100.92, 112.07, and 116.15 ng/mL, respectively; AUC_0-inf_ was 1209.38, 1424.76, 1797.95, and 1832.67 ng hr/mL, respectively. Regarding urine PK parameters, the fraction of excreted dotinurad in urine from time zero to 48 h (fe_0-48_) was 0.82–1.51% in the overall groups.

**Table 2:** PK parameters of dotinurad in plasma and urine

PK parameter	Elderly males (*n* = 6)	Young males (*n* = 6)	Elderly females (*n* = 6)	Young females (*n* = 6)	*P* value			*P* value (body weight correction)		
					Age^a^	Gender^b^	Total^c^	Age^a^	Gender^b^	Total^c^
	Mean ± SD	Mean ± SD	Mean ± SD	Mean ± SD	Male/Female	Elderly/Young	Age/Gender	Male/Female	Elderly/Young	Age/Gender
*C*_max_ (ng/mL)	93.30 ± 16.07	100.92 ± 21.20	112.07 ± 12.66	116.15 ± 26.67	0.558/0.897	0.045*/0.358	0.621/0.050	–	0.390/0.160	–/0.795
*T*_max_ (hr)	2.00 ± 0.63	2.17 ± 0.75	2.17 ± 0.75	2.83 ± 0.98	0.687/0.217	0.687/0.217	0.213/0.213	–	–	–
*T*_1/2_ (hr)	9.28 ± 1.05	10.31 ± 1.27	10.92 ± 1.19	10.47 ± 0.31	0.156/0.386	0.029*/0.775	0.546/0.050	–	–	–
AUC_0–t_ (ng hr/mL)	1170.41 ± 277.44	1365.15 ± 232.50	1703.20 ± 320.14	1750.33 ± 328.96	0.224/0.827	0.012*/0.056	0.382/0.001*	–	0.064/0.754	–/0.133
AUC_0–inf_ (ng hr/mL)	1209.38 ± 290.88	1424.76 ± 242.34	1797.95 ± 357.84	1832.67 ± 345.74	0.199/0.874	0.011*/0.053	0.383/ < 0.001*	–	0.055/0.790	–/0.112
CL_tot_/F (L/hr)	0.870 ± 0.220	0.723 ± 0.152	0.574 ± 0.112	0.565 ± 0.125	–	–	–	–	–	–
kel (1/hr)	0.0756 ± 0.0098	0.0681 ± 0.0089	0.0642 ± 0.0080	0.0663 ± 0.0019	–	–	–	–	–	–
Vd/F (L)	11.42 ± 1.86	10.81 ± 3.00	8.91 ± 0.98	8.51 ± 1.76	–	–	–	–	–	–
MRT_0-t_ (hr)	11.33 ± 1.26	12.48 ± 1.02	12.98 ± 0.84	13.05 ± 0.28	–	–	–	–	–	–
Ae_0-48_ (μg)	9.51 ± 4.31	15.08 ± 4.30	8.20 ± 2.46	10.50 ± 4.75	–	–	–	–	–	–
fe_0–48_ (%)	0.9509 ± 0.4314	1.5081 ± 0.4300	0.8200 ± 0.2456	1.0498 ± 0.4748	–	–	–	–	–	–

^a^Elderly males vs. young males/elderly females vs. young females

^b^Elderly males vs. elderly females/ young males vs. young females

^c^Elderly vs. young/males vs. females

*P  < 0.05 (*t* test: *C*_max_, AUC_0–t_, and AUC_0-inf_ were compared after logarithmic transformation)

On comparing plasma PK parameters of dotinurad, there was no significant difference between elderly and young groups in the main PK parameters, including *C*_max_, *T*_max_, *T*_1/2_, AUC_0-t_, and AUC_0-inf_. On the other hand, significant differences were observed in *C*_max_, *T*_1/2_, AUC_0-t_, and AUC_0-inf_ between elderly male and elderly female groups. However, no significant difference was observed in any PK parameter between young male and young female groups.

Tables 3 and 4 show the PK parameters of major metabolites of dotinurad in plasma and urine. In each group, the concentrations of glucuronide and sulfate conjugates in plasma were very low. Regarding urine PK parameters, fe_0-48_ ranged 32.70–40.35% for the glucuronide conjugate and 12.72–15.90% for the sulfate conjugate.

**Table 3:** PK parameters of glucuronide conjugate in plasma and urine

PK parameter	Elderly male (*n* = 6)		Young males (*n* = 6)		Elderly female (*n* = 6)		Young female (*n* = 6)	
	*n*	Mean ± SD	*n*	Mean ± SD	*n*	Mean ± SD	*n*	Mean ± SD
*C*_max_ (ng/mL)	5	1.64 ± 0.33	3	N.C	6	1.48 ± 0.25	3	N.C
*T*_max_ (hr)	5	1.80 ± 0.84	3	N.C	6	2.17 ± 0.75	3	N.C
*T*_1/2_ (hr)	4	11.76 ± 3.72	1	N.C	5	11.47 ± 3.40	0	N.C
AUC_0–t_ (ng hr/mL)	5	7.48 ± 2.84	3	N.C	6	8.16 ± 4.45	3	N.C
AUC_0–inf_ (ng hr/mL)	4	25.58 ± 2.83	1	N.C	5	27.04 ± 8.36	0	N.C
CL_tot_/F (L/hr)	4	39.443 ± 4.177	1	N.C	5	39.675 ± 11.164	0	N.C
kel (1/hr)	4	0.0646 ± 0.0247	1	N.C	5	0.0656 ± 0.0224	0	N.C
Vd/F (L)	4	655.78 ± 161.84	1	N.C	5	616.26 ± 93.47	0	N.C
MRT_0–t_ (hr)	5	3.41 ± 0.75	3	N.C	6	3.83 ± 1.29	3	N.C
Ae_0–48_ (μg)	6	492.87 ± 87.02	6	601.93 ± 63.61	6	516.54 ± 59.10	6	487.81 ± 77.34
fe_0–48_ (%)	6	33.0410 ± 5.8340	6	40.3523 ± 4.2646	6	34.6284 ± 3.9619	6	32.7018 ± 5.1850

*N.C.* Summary statistics were not calculated because calculation was not possible in at least half of the patients

**Table 4:** PK parameters of sulfate conjugate in plasma and urine

PK parameter	Elderly males (*n* = 6)		Young males (*n* = 6)		Elderly females (*n* = 6)		Young females (*n* = 6)	
	*n*	Mean ± SD	*n*	Mean ± SD	*n*	Mean ± SD	*n*	Mean ± SD
*C*_max_ (ng/mL)	6	2.37 ± 0.84	6	1.83 ± 0.45	6	2.65 ± 0.82	6	1.95 ± 0.65
*T*_max_ (hr)	6	2.67 ± 1.51	6	2.00 ± 0.89	6	2.50 ± 1.87	6	2.33 ± 0.82
*T*_1/2_ (hr)	4	7.53 ± 1.87	4	11.22 ± 8.15	5	10.38 ± 10.03	5	7.36 ± 4.20
AUC_0–t_ (ng hr/mL)	6	13.68 ± 7.88	6	7.73 ± 4.14	6	17.55 ± 6.89	6	11.58 ± 4.49
AUC_0–inf_ (ng h/mL)	4	30.78 ± 7.60	4	25.93 ± 13.98	5	34.21 ± 13.79	5	23.86 ± 5.87
CL_tot_/F (L/hr)	4	33.964 ± 8.001	4	45.822 ± 18.736	5	33.476 ± 13.575	5	44.222 ± 11.954
kel (1/hr)	4	0.0976 ± 0.0303	4	0.0880 ± 0.0512	5	0.1018 ± 0.0493	5	0.1243 ± 0.0720
Vd/F (L)	4	358.37 ± 83.19	4	584.78 ± 159.82	5	398.59 ± 218.71	5	421.92 ± 154.26
MRT_0–t_ (hr)	6	4.05 ± 1.15	6	3.80 ± 1.01	6	4.81 ± 0.80	6	5.11 ± 1.19
Ae_0–48_ (μg)	6	194.55 ± 39.31	6	184.19 ± 47.84	6	170.37 ± 38.36	6	155.58 ± 21.75
fe_0–48_ (%)	6	15.9008 ± 3.2127	6	15.0542 ± 3.9101	6	13.9246 ± 3.1349	6	12.7163 ± 1.7776

*N.C.* Summary statistics were not calculated because calculation was not possible in at least half of the patients

### Pharmacodynamics

**Fig. 2:**
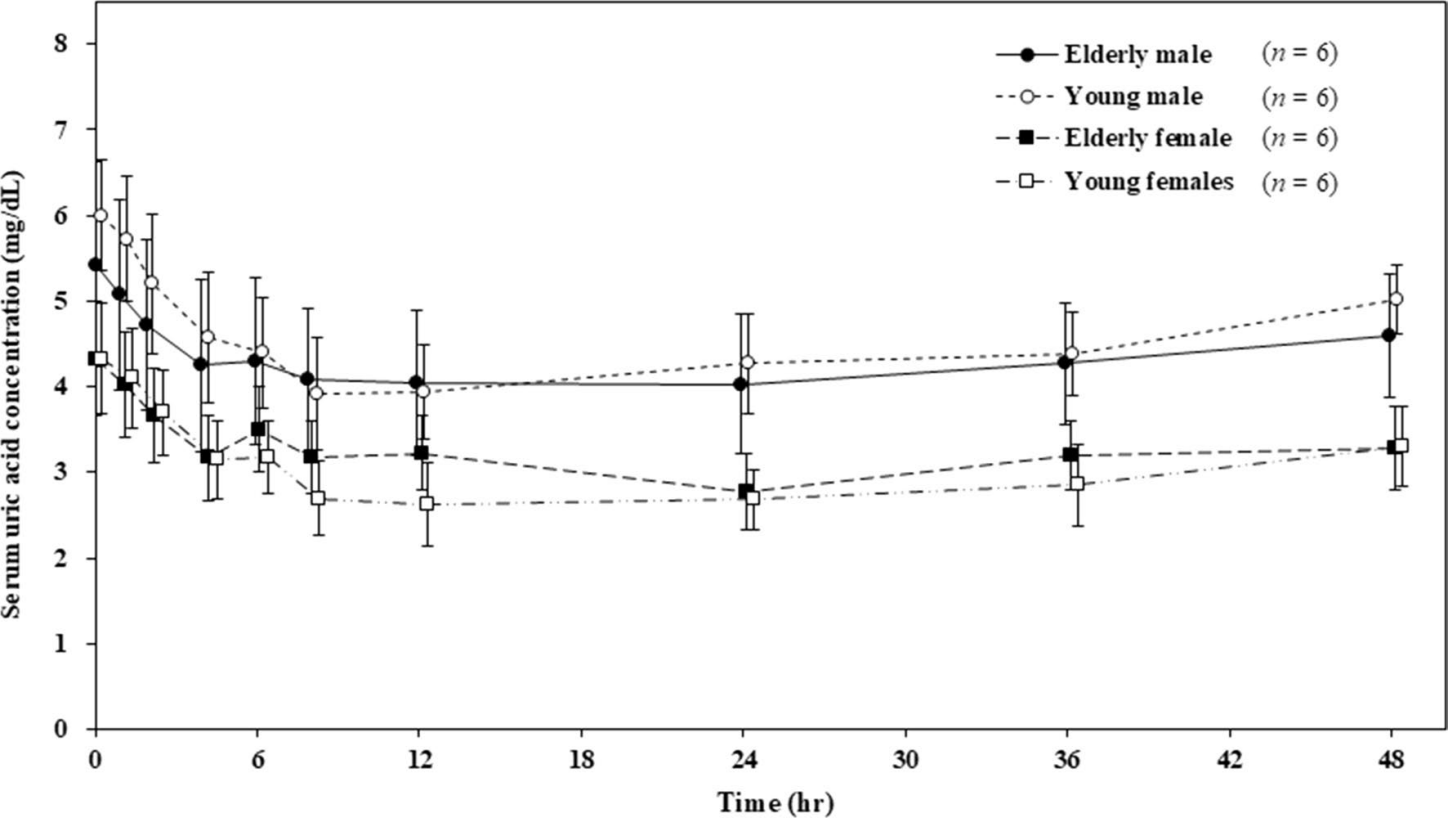
Time course of serum uric acid concentrations. Error bars indicate standard deviation

Figure 2 shows the time course of the serum uric acid concentrations in each group. In all the groups, the serum uric acid concentrations decreased within an hour after dotinurad administration. Furthermore, the serum uric acid concentrations were lowest between 8 and 24 h after administration and then increased. However, the serum uric acid concentration after 48 h from administration was slightly lower than those before administration. Table 5 shows the serum and urine PD parameters. In the elderly male, young male, elderly female, and young female groups, ΔEC_max_ was –1.47, –2.13, –1.57, and –1.77 mg/dL, respectively, and ΔAUEC_0-48_ was – 56.98, – 76.40, – 56.27, and – 68.15 mg hr/dL, respectively. In all the groups, dotinurad increased CL_R_, FE, and Ae_0-24_ approximately twice as much as before administration. Moreover, Ae_24-48_ decreased and recovered to the same level as before administration.

**Table 5:** PD parameters of uric acid in serum and urine

PD parameter	Elderly male (*n* = 6)	Young male (*n* = 6)	Elderly female (*n* = 6)	Young female (*n* = 6)	*P* value		
	Mean ± SD	Mean ± SD	Mean ± SD	Mean ± SD	Age^a^(male/female)	Gender^b^(elderly/young)	Total^c^(age/gender)
ΔEC_max_ (mg/dL)	– 1.47 ± 0.49	– 2.13 ± 0.49	– 1.57 ± 0.34	– 1.77 ± 0.34	0.040*/0.334	0.689/0.164	0.021*/0.501
ΔAUEC_0-48_ (mg hr/dL)	– 56.98 ± 22.30	– 76.40 ± 23.57	– 56.27 ± 13.75	– 68.15 ± 13.49	0.173/0.162	0.949/0.474	0.047*/0.586
Ae_–24–0_ (mg)	489.58 ± 103.70	507.32 ± 72.37	391.84 ± 66.16	385.16 ± 75.58	–	–	–
Ae_0-24_ (mg)	849.77 ± 118.02	1058.84 ± 68.56	745.67 ± 71.38	768.89 ± 106.12	–	–	–
Ae_24-48_ (mg)	419.30 ± 57.37	453.78 ± 76.11	398.20 ± 50.22	383.20 ± 39.24	–	–	–
Ae_0-48_ (mg)	1269.07 ± 168.33	1512.62 ± 115.34	1143.87 ± 118.60	1152.09 ± 134.95	–	–	–
CL_R–24–0_ (mL/min)	6.41 ± 1.45	5.94 ± 1.06	6.39 ± 1.43	6.25 ± 1.30	–	–	–
CL_R0-24_ (mL/min)	14.32 ± 2.20	17.35 ± 2.59	16.46 ± 3.12	18.49 ± 1.90	–	–	–
CL_R24-48_ (mL/min)	6.91 ± 1.27	7.05 ± 1.36	9.07 ± 1.92	9.22 ± 1.24	–	–	–
FE_–24–0_ (%)	6.10 ± 1.05	4.24 ± 0.55	6.02 ± 1.09	5.42 ± 0.87	0.003*/0.313	0.901/0.019*	0.005*/0.248
FE_0-24_ (%)	14.00 ± 1.48	13.07 ± 2.52	15.11 ± 2.47	14.85 ± 1.85	0.453/0.837	0.368/0.194	0.506/0.100
FE_24-48_ (%)	6.78 ± 0.81	5.52 ± 0.96	7.74 ± 1.46	7.37 ± 1.56	0.035*/0.676	0.187/0.034*	0.171/0.013*
FE_0-24_/FE_–24–0_	2.33 ± 0.33	3.12 ± 0.68	2.52 ± 0.12	2.76 ± 0.29	0.029*/0.082	0.223/0.265	0.006*/0.674
FE_24-48_/FE_–24–0_	1.13 ± 0.14	1.31 ± 0.23	1.29 ± 0.12	1.36 ± 0.18	0.114/0.434	0.056/0.705	0.083/0.172

^a^Elderly males vs. young males/elderly females vs. young females

^b^Elderly males vs. elderly females/young males vs. young females

^c^Elderly vs. young/males vs. females

*P < 0.05 (*t* test for mean difference)

On comparing PD parameters for elderly versus young subjects, significant differences were observed in ΔEC_max_, FE_–24–0_, FE_24-48_, and FE_0-24_/FE_–24–0_ between elderly male and young male groups, whereas no significant difference was observed in ΔAUEC_0-48_ between these groups and in any parameter between female groups. On comparing for male versus female subjects, there was no significant difference in any PD parameter between elderly male and elderly female groups. However, significant differences were observed for FE_–24–0_ and FE_24-48_ between young groups.

### Safety

No discontinuations occurred due to a serious adverse event (SAE) or AE. The incidence of AEs was 50% (4 events in 3 subjects) in elderly male group, 50% (3 events in 3 subjects) in young male group, 50% (4 events in 3 subjects) in elderly female group, and 83.3% (12 events in 5 subjects) in young female group. All events were mild and non-serious and recovered without treatment. The following ADRs were observed: urinary casts present (one event in one subject) in elderly male group; supraventricular extrasystoles (one event in one subject) and faeces hard (one event in one subject) in elderly female group; and white blood cells urine positive (four events in three subjects), urinary sediment present (two events in two subjects), and bacterial test positive (one event in one subject) in young female group.

## Discussion

For the main PK parameters (*C*_max_, *T*_max_, *T*_1/2_, AUC_0-t_, and AUC_0-inf_) of dotinurad, there were no significant differences between elderly and young groups, whether male or female. When male and female subjects were compared, significant differences were observed in *C*_max_, *T*_1/2_, AUC_0-t_, and AUC_0-inf_ between elderly groups. However, significant differences in these PK parameters could not be detected by adjusting each parameter for body weight. These differences between genders were, therefore, thought to be due to differences in body weight (Table 2). In contrast, no significant difference in these PK parameters was observed between young subject groups.

In this study, dotinurad showed a serum uric acid lowering effect and increased amount of urinary uric acid excretion, regardless of age and gender. Comparing PD parameters for age, significant differences were observed in ΔEC_max_, FE_–24–0_, FE_24-48_, and FE_0-24_/FE_–24–0_ between elderly male and young male groups. For these PD parameters, we assumed effects of the difference in values before administration (FE_–24–0_). On comparing male and female, no significant differences were observed in elderly groups. Significant differences were observed in FE_–24–0_ and FE_24-48_ in young groups; however, the time course of percent change in serum uric acid concentration was relatively close between young male and young female groups. Gender differences have been reported in serum uric acid concentration [9]. Usually, its values are lower in females; however, any gender difference has been reported to decrease after menopause. In this study, the serum uric acid concentration before administration was lower in females than that in males. Although no significant differences were observed in PD parameters between elderly males and females, a significant difference was observed in the fraction of excreted uric acid in urine between young males and females. We speculate that one cause of this difference is an increase in uric acid clearance due to female hormones like estrogen [9]. The percent change in serum uric acid concentration was relatively close for young male and female groups; therefore, the effect of gender on the serum uric acid lowering effect was considered small.

Regarding safety, the incidence of AEs tended to be greatest in young females. However, the severity of all AEs was mild, and no SAE or discontinuations due to AEs were observed in this study. Therefore, no safety concerns were observed regardless of age or gender.

In conclusion, no meaningful effect of age or gender was observed in the PK and PD of dotinurad in healthy adults. Moreover, regarding clinical safety no problematic AE or ADR was observed. These findings suggest that dotinurad can be administered to all the patients regardless of gender, and without dose adjustment in elderly patients.
